# Biological therapy management from the initial selection of biologics to switching between biologics in severe asthma

**DOI:** 10.5578/tt.20239910

**Published:** 2023-03-10

**Authors:** İ.Y. Gülden, G.P. Çetin, B. Arslan, S. Şeker, H.E.B. Yılmaz, E.A. Yapıcı, S. Köylüce, E. Açar

**Affiliations:** 1 Division of Immunology and Allergy, Department of Chest Diseases, Erciyes University Faculty of Medicine, Kayseri, Türkiye

**Keywords:** Severe asthma, biologics, mepolizumab, omalizumab, dupilumab, reslizumab, benralizumab, Ağır astım, biyolojik ajanlar, mepolizumab, omalizumab, dupilumab, reslizumab, benralizumab

## Abstract

**ABSTRACT:**

**Biologics for the treatment of severe asthma: Current status report 2023:**

Severe asthma is associated with increased use of healthcare services, significant deterioration in the quality of life, and high disease and economic burden on patients and societies. Additional treatments are required for severe forms of asthma. Biological agents are recommended for the treatment of severe asthma. In this current status report, we aimed to evaluate the efficacy, effectiveness, and safety data of approved biologics; omalizumab, mepolizumab, reslizumab, benralizumab, dupilumab, and tezepelumab, in the treatment of severe asthma and appropriate patient profiles for these biologics. Pubmed and Cochrane databases based on randomized controlled trials, posthoc analyses, meta-analyses, and real-life studies examining the efficacy and effectiveness of biologics in severe asthma were searched, and the results of these studies on important asthma outcomes were reviewed. Existing studies have shown that all the approved biologic agents targeting cells, receptors, and mediators involved in type 2 inflammation in the bronchial wall in severe asthma significantly reduce asthma exacerbations, reduce the need for oral corticosteroids, and improve asthma control, quality of life, and pulmonary functions. Characterizing the asthma endotype and phenotype in patients with severe asthma and determining which treatment would be more appropriate for a particular patient is an essential step in personalized treatment.

## Abstract (tr)

**Ağır astımda biyolojik ajanların ilk seçiminden biyolojikler arasında geçişe kadar biyolojik tedavi yönetimi:**

Bu derlemede ağır astımda, başlangıctaki biyolojik ajan seçiminden biyolojikler arasındaki gecişe kadar olan tedavi yönetiminin detaylı bir şekilde gözden geçirilmesi amaçlanmıştır. Ağır astımda biyolojik tedavi yönetimi için sistematik olmayan bir inceleme yapıldı. Klinik özelliklere, biyobelirteçlere ve önceki çalışmalardan elde edilen süper yanıt verme kriterlerine dayalı olarak biyolojik ajanın seçilmesi, çoğu hastada yeterli klinik etkinlikle sonuçlanabilir. Öte yandan, seçim ne kadar dikkatli yapılırsa yapılsın, bazı hastalarda suboptimal klinik yanıt veya yanıt alınamaması nedeniyle ilacın kesilmesi gerekebilir. Bu, farklı bir biyolojik tedaviye geçme ihtiyacına neden olabilir. Biyolojik ajanlarla astımı kontrol altına alınan hastaların biyolojik tedavisine ne kadar devam edileceği, tedavinin hangi kriterlere göre sonlandırılacağı belirsizliğini korumaktadır. Biyolojik ajanlarla uzun vadede iyi yanıt alınan hastalarda bu ilaçlar kesildiğinde astım kontrolünün bozulabileceği, bazı hastalarda ise astım kontrolünün devam ettiği gösterilmiştir. Bu nedenle, belirli bir hasta grubunda biyolojik ilaçların kesilmesi geçerli bir strateji olabilir. Klinisyenler, her bir biyolojikten en fazla fayda sağlayacak hastaları belirlemek için tüm prediktif faktörleri en iyi şekilde kullanmalıdır. Yanıt değerlendirmesi için yeterli süre geçtikten sonra önceden tanımlanmış bir yanıt kriterini karşılamayan ve bir veya daha fazla alternatif biyolojik ajan için uygun olan hastalara başka bir biyolojik ajana geçme fırsatı sunulmalıdır. Ağır astımda kullanılan ve tedaviye iyi yanıt veren biyolojik ajanların ne zaman kesileceği konusunda fikir birliği yoktur. Erken sonlandırmalarda astım kontrolü bozulabileceği için en az beş yıl devam etmek mantıklı bir yaklaşım gibi görünmektedir

## Introduction

With the development of biologics for severe asthma (SA) treatment in recent years, asthma management has improved. There are six monoclonal antibodies (mAb) approved by the U.S. Food and Drug Administration (FDA) and/or the European Medicines Agency (EMA) for the treatment of asthma. These are omalizumab (anti-IgE), mepolizumab (anti-IL5), reslizumab (anti-IL5), benralizumab (anti-IL5Rα), dupilumab (anti-IL4Rα), and tezepelumab (antiTSLP), in order of approval ( 1 , 2 , 3 , 4 , 5 , 6

The first choice in SA can be complicated by the increase in the number of mAbs, the fact that a patient may have indications for more than one mAb, and the lack of head-to-head trials to inform clinical decisions.

To the best of our knowledge, well-known genetic risk factors for pulmonary fibrosis, TERT, and MUC5B mutations, have not been evaluated in individuals with CAPF. The present study aims to explore the role of mutations of TERT and MUC5B genes in the development of CAPF.

In addition, no matter how much attention is paid when starting the first mAb, in some patients, partial or no response may result in the discontinuation of the biologic. This may lead to the need to switch to a different mAb. In this review, the initial mAb choice, markers that can predict treatment response, treatment response criteria, duration of continuation, discontinuation of treatment, and switching between mAbs are discussed in detail.

### Choosing the Initial mAb

In clinical practice; patient age, severity, phenotype, biomarkers, therapeutic goals, comorbidities, and cost should all be considered when choosing the initial mAb in asthma ( 7 ). Furthermore, drug administration options, expected benefits of mAbs, and mAb safety profiles should be shared with patients and decided collaboratively ( 7 ).

Since all the biologics used in asthma are effective in type 2 (T2) asthma (tezepelumab can also be effective regardless of the T2 endotype), it should first be determined whether the patient has the T2 endotype. T2 inflammation, characterized by increased blood and airway eosinophils and increased specific IgE and FeNO levels, has been demonstrated in approximately 70-80% of patients with SA ( 8 ). The presence of any of the following while receiving high-dose ICS or mOCS in SA suggests refractory T2 inflammation ( 9 ): • Blood eosinophils≥ 150 cells/µL • FeNO≥ 20 ppb • ≥2% sputum eosinophil • Clinically consistent asthma caused by allergens.

Indications for mAb in SA according to GINA report are shown in Table 1 ( 9 ):

Clinical phase studies with these biologics, FDA/EMA approvals, recommendations of international guidelines, and socioeconomic conditions of countries are evaluated by local regulators, and approvals for use and/or reimbursement are granted

**Table 1:** Biologics as add-on therapy in severe asthma

Biologic	Eligibility criteria
Anti-IgE (omalizumab)	· Sensitivity to inhalant allergens (with skin prick test or specific IgE) and · Serum total IgE and body weight within the range of the dosing chart and · At least one asthma exacerbation within the last year
Anti-IL5/Anti-IL5Rα (mepolizumab, reslizumab, benralizumab)	· Elevated blood eosinophils by locally specific criteria (based on the clinical phase studies, blood eosinophil≥ 400 cell/µL for reslizumab, ≥300 cells/µL for benralizumab, and ≥300 cells/µL within the last year or ≥150 cells/µL at baseline for mepolizumab and · At least one asthma exacerbation within the last year
Anti-IL4Rα (dupilumab)	· Blood eosinophils ≥150 and ≤1500 cells/µL, or FeNO≥ 25 ppb, or OCS-dependent asthma and · At least one asthma exacerbation within the last year
Anti-TSLP (tezepelumab)	At least one asthma exacerbation within the last year

IgE: Immunoglobulin E, OCS: Oral corticosteroid, TSLP: Thymic stromal lymphopoietin, FeNO: Fractional exhaled nitric oxide.

in countries based on these considerations. In other words, the final use indication decision is made by the scientific boards and reimbursement institutions of the respective countries. Therefore, factors such as the availability of biologics in countries, their licensing, and reimbursement status by local regulators are the most critical factors affecting prescription decisions. Other than clinical and biomarkers, some additional factors shown below that can guide mAb selection in asthma should also be considered ( 10 ). These factors are given in Table 2 ( 1 , 2 , 3 , 4 , 5 , 6 , 7 , 11 , 12 , 13 , 14 , 15 , 16 , 17 , 18 , 19 , 20 , 21 , 22 , 23 , 24 , 25 , 26 , 27 , 28 , 29 , 30 , 31 , 32 , 33 , 34 , 35 , 36 , 37 , 38 , 39 , 40 , 41 , 42 , 43 , 44 , 45 , 46 , 47 , 48 ).

#### Markers Used to Predict the Response to Biologics in Severe Asthma

The initial biologic treatment should be chosen very carefully. The markers used to predict response should be evaluated to determine which biologic has the potential to benefit more in patients with multiple mAb indications ( 7 ).

##### Markers used to predict anti-IgE treatment response

Although studies show that omalizumab treatment is more effective in severe atopic asthma in SA patients with high blood eosinophil and FeNO levels, there are also studies showing that it is effective regardless of the baseline characteristics and biomarker levels of the patients ( 49 , 50 , 51 ).

In the EXTRA study by Hanania et al., it was shown that the treatment efficacy of omalizumab was higher at high baseline biomarker levels (blood eosinophil, FeNO, and periostin) than at low biomarker levels. This indicates that blood eosinophils, FeNO, and periostin may be predictive biomarkers that can be used to determine treatment response to omalizumab in atopic asthma ( 49 ). On the contrary, another study reported that there was no significant difference between the group with high biomarker levels and the group with low biomarker levels in terms of omalizumab treatment response (blood eosinophil< 300 cells/µL or ≥300 cells/µL and FeNO< 25 ppb or ≥25 ppb) (51). However, this study was criticized for the absence of a placebo arm. In addition, baseline biomarkers were associated with improvement in asthma control testing (ACT) and lung function, but the extent of this improvement was not clinically relevant (hospitalization, number of asthma exacerbations).

**Table 2:** Biological agents used in severe asthma treatment ( 1 , 2 , 3 , 4 , 5 , 6 , 7 , 11 , 12 , 13 , 14 , 15 , 16 , 17 , 18 , 19 , 20 , 21 , 22 , 23 , 24 , 25 , 26 , 27 , 28 , 29 , 30 , 31 , 32 , 33 , 34 , 35 , 36 , 37 , 38 , 39 , 40 , 41 , 42 , 43 , 44 , 45 , 46 , 47 , 48 )

	Biologic	Age	Administration and forms	Dosage	Other indications	Cost-effectiveness	Safety
Anti-IgE	Omalizumab	≥6	SC, prefilled syringe	75-375 mg (every 2-4 wk.) According to body weight and serum total IgE	CSU CRSwNP	SAA: Cost-effective	Similar to placebo
Anti-IL-5	Mepolizumab	≥6	SC, prefilled syringe, autoinjector pen	Adults and adolescents: 100 mg every four wk. Children aged 6-11: 40 mg every four wk.	CRSwNP EGPA HES (adults)	Specific subgroups for SA	Similar to placebo
Anti-IL-5	Reslizumab	≥18	IV infusion	3 mg/kg every four wk.		Not cost-effective	Similar to placebo
Anti-IL-5R	Dupilumab	≥6	SC, prefilled syringe, autoinjector pen	The initial dose of 400 mg followed by 200 mg every two wk., The initial dose of 600 mg, followed by 300 mg every two wk.	CRSwNP Atopic dermatitis	Not cost-effective	Similar to placebo
Anti-TSLP	Tezepelumab	≥12	SC, prefilled syringe	210 mg every four wk.		NA	Similar to placebo

TSLP: Thymic stromal lymphopoietin, SC: Subcutaneous, IV: Intravenous, wk.: Week, CSU: Chronic spontaneous urticaria, CRSwNP: Chronic rhinosinusitis with nasal polyps, HES: Hypereosinophilic syndrome, EGPA: Eosinophilic granulomatosis with polyangiitis, SAA: Severe allergic asthma, SA: Severe asthma, SEA: Severe eosinophilic asthma, NA: Not applicable.

In a real-life retrospective study including patients with atopic asthma, omalizumab efficacy was similar in high and low eosinophil subgroups ( 52 ). However, when we examine this study in detail, we see that atopic asthmatics are not homogeneous, especially in adults. It is seen that nasal polyps (NP) are present in approximately 1/3 of adults but not at all in the pediatric age group. At the time of starting omalizumab, 34% of adults had OCS use, while this rate was 2% in children. As a result, pediatric atopic asthma patients formed a more homogeneous group, and the rate of those with eosinophils> 300 cells/µL was also relatively high. Atopic asthmatics appear to have a more heterogeneous clinical/inflammatory phenotype in adults. Treatment response rates in the pediatric age group with more homogeneous atopic asthma seem much better in patients with eosinophils> 300 cells/µL than in adults.

The use of omalizumab in asthma patients with chronic rhinosinusitis with nasal polyp (CRSwNP) has been called into question. Two randomized phase 3 trials demonstrated the efficacy of omalizumab in treating CRSwNP. In these phase studies that led to the approval of omalizumab for atopy-independent nasal steroid-resistant CRSwNP, the majority (>90%) of patients with CRSwNP were mild-to-moderate asthmatics (53). Therefore, additional studies must confirm omalizumab’s efficacy in SA with CRSwNP. Considering the studies on this subject, the GINA report states that the cut-off values for blood eosinophils and FeNO may affect the omalizumab response. Table 3 shows which conditions warrant anti-IgE treatment, mainly in uncontrolled SA with sensitivity to perennial aeroallergens.

###### Markers used to predict Anti-IL5/IL5Rα treatment response

The predictive factors for a good response to biologics are shown in Table 3 ( 9 , 54 , 55 , 56 , 57 , 58 , 59 , 60 , 61 , 62 , 63 , 64 , 65 , 66 , 67 , 68 , 69 ). Real-life studies can help determine the profile of patients who respond well to treatment and the impact of comorbidities that may have been excluded in RCTs. A two year real-life follow-up study by Eger K et al. included patients with SA who were started on an anti-IL5/IL5Rα biologic (mepolizumab, reslizumab, benralizumab). The patients who were super-responders to anti-IL5 treatment were shown to have a shorter duration of asthma, a higher FEV_1_ level, and although not statistically significant, were also associated with

**Table 3:** The predictive factors for a good response to biologics

Biologic	Criteria for better response to treatment
Anti-IgE (omalizumab)	· Blood eosinophil≥ 260 cells/µL · FeNO≥ 20 ppb · Allergen-driven symptoms · Childhood-onset asthma
Anti-IL5/Anti-IL5Rα (mepolizumab, reslizumab, benralizumab)	· Higher blood eosinophils (strong predictor) · More exacerbations in the previous year (good predictor) · Adult-onset asthma · Nasal polyps · Maintenance OCS · Lower pulmonary functions (predictive FEV1< 65%)
Anti-IL4Rα (dupilumab)	· Higher blood eosinophils (strong predictor) · Higher FeNO (strong predictor)
Anti-TSLP (tezepelumab)	· Higher blood eosinophils (strong predictor) · Higher FeNO (strong predictor)

IgE: Immunoglobulin E, OCS: Oral corticosteroid, FeNO: Fractional exhaled nitric oxide.

adult-onset asthma, absence of NPs, and low body mass index. The remarkable point in this study was that although there was no statistical significance in super-responder patients, there was an association with the absence of NP ( 70 ). However, studies have shown that the presence of comorbid NP predicts a good response to anti-IL5/IL5Rα mAbs in SA ( 55 , 69 , 71 , 72 ). In addition, in the GINA report, NP is included in the criteria for a good response to anti-IL5/ IL5Rα in patients with SEA ( 9 ). Therefore, these results differ from other studies and the GINA report ( 9 ).

However, it is unclear how frequently these predictive factors are used in clinical practice. Indeed, studies show that in clinical practice, clinicians consider markers and comorbidities that have the potential to predict treatment response when selecting the initial biologic ( 71 , 73 ). Table 3 presents potential markers that can predict a good response to anti-IL5/IL5Rα therapy in T2 SA based on the findings of all clinical and real-life studies on this issue in the GINA report.

####### Markers used to predict anti-IL4Rα (dupilumab) treatment response

The therapeutic efficacy of dupilumab is greater in patients with higher baseline blood eosinophil counts (more reduction in exacerbations in those with >150 cells/µL, further improvement in FEV_1_ in those with >300 cells/µL) and high FeNO levels (greater improvement in FEV_1_ in those >50 ppb) ( 9 , 74 ) (Table 3).

The FDA approved dupilumab for the treatment of moderate-to-severe atopic dermatitis (AD) before asthma. Dupilumab can be the first choice mAb in atopic eosinophilic SA phenotype with moderate to severe AD if blood eosinophils are ≥300 cells/µL or FeNO is ≥25 ppb. Due to its efficacy in AD and CRSwNP, dupilumab should be considered for the treatment of patients with uncontrolled SA accompanied by these comorbidities ( 75 , 76 ). In the GINA report, potential markers that may predict a good response to dupilumab are listed as higher blood eosinophils (strong predictor) and higher FeNO (strong predictor) levels ( 9 ).

######## Markers used to predict anti-TSLP (tezepelumab) treatment response

Tezepelumab, recently approved for use in SA, has been approved for both T2 and non-T2 asthma endotypes ( 77 , 78 ). In the clinical phase 2 study conducted by Corren et al., a significant decrease in the number of annual exacerbations and an increase in FEV_1_ were found in the patient groups receiving tezepelumab, independent of the baseline blood eosinophil count, compared to the placebo group ( 79 ). In the post-hoc analysis of this study, tezepelumab was shown to reduce exacerbations and T2 inflammatory biomarkers in patients with and without CRSwNP, supporting its efficacy in a large patient population with SA ( 80 ).

In the SOURCE study evaluating the OCS-reducing effect of tezepelumab in OCS-dependent patients, no significant improvement was demonstrated in OCS reduction with tezepelumab versus placebo ( 81 ).

In the GINA report ( 9 ), potential predictors for a good response to treatment are listed as high blood eosinophils (strong predictive) and high FeNO (strong predictive). Although there is no reference in GINA for the high predictive value of these biomarkers, we believe that the NAVIGATOR study may be a source that can support this recommendation ( 82 ) (Table 3). More studies, including real-life studies, are needed to identify potential predictors.

######### The Time and Response Criteria for the Initial Response to Biologics

At the beginning of the treatment, the response to mAbs is evaluated using clinical and biological indicators. While the current GINA report for SA treatment recommends waiting four months before assessing a patient's mAb treatment response, the exact time to determine whether the patient is responding or not has not yet been clearly defined. Another controversial point is that the initial criteria for determining the response to biologics are not standardized. Therefore, using different “response” or “non-response” criteria in the initial assessment of the mAb response may also result in different response rates.

Asthma Control Test (ACT), Asthma Control Questionnaire (ACQ), and GINA symptom control categorical scores, as well as using the Global Evaluation of Treatment Effectiveness (GETE) scoring, are some of the parameters that are typically compared to baseline during treatment response evaluations at the 16th week of biologic treatment.

Some studies of the time and response criteria of the initial evaluation of the response to treatment with anti-IgE, anti-IL5/anti-IL5Rα, and anti-IL4Rα treatments are shown in Table 4 ( 61 , 83 , 84 , 85 , 86 , 87 , 88 , 89 , 90 , 91 , 92 ).

The studies show that the initial evaluation criteria for response to biologics are not standardized. As a result, it is recommended that the patients complete at least 4-6 months of treatment with biologics to assess the first response. If no asthma response is received at the desired level, the time can be extended by 6-12 months ( 9 ). Our opinion is in the initial assessment (usually at 16t^th^ week): • Notable change in ACT or ACQ without an increase in the number of exacerbations compared to the pre-biological 16 weeks, • In OCS-dependent patients, the OCS dose can be reduced without an increase in the number of exacerbations and without deterioration of ACT/ ACQ compared to the pre-biological 16 weeks. • If the dose of OCS cannot be reduced, a significant improvement in ACT/ACQ scores without an increase in the number of exacerbations compared to the pre-biological 16 weeks is considered a response to treatment, and mAb can be continued. One of the important points here is that the reduction of OCS therapy in patients with OCSdependent asthma is accepted as the most reliable indicator for evaluating the clinical success of these treatments ( 93 ). However, in this early initial evaluation of the response to mAbs, in case of significant improvement in ACT/ACQ scores even though the dose of OCS cannot be reduced [this may be included in the definition of treatment that can be extended to 6-12 months in case of failure to achieve the desired level of response (suboptimal response) specified in GINA report]. We think that if the dose of OCS cannot be reduced after one year, that is, if the suboptimal response continues, discontinuation of the biologic should be considered.

########## Time to and Criteria for Discontinuing Treatment in Patients with Good Response to Biologicals, and the Effectiveness of Biologics After Treatment Termination

########### Time to discontinue treatment

With the advent of T2-targeted biologics, some SA has become a “controllable” state. However, it is unclear how long mAb treatment should be continued in patients whose asthma is controlled by these biologics and under what conditions it should be discontinued. There is no consensus on this issue in the literature ( 8 ). It has been shown that in patients with a long history of good response to biologics, asthma control may be impaired when biologics are discontinued, while it may persist in others. Therefore, discontinuation of mAbs may be a viable strategy in a particular patient group ( 94 , 95 , 96 ).

**Table 4:** Initial response criteria for biological agents

Biologic	Study	First evaluation time of response to biologic	Response criteria
Omalizumab	Kucharczyk A et al., 2020, ( 83 )	16 wk.	· GETE scale: Very good or good response to treatment and · A decrease in the annual exacerbation rate (any reduction) and · At least two of the following: a. increase in miniAQLQ by >0.5 points b. decrease in ACQ-7 by >0.5 points c. any reduction in the OCS dose.
Omalizumab	Kupryś-Lipińska I et al., 2016, ( 84 )	16 wk.	· GETE scale: Very good or good response to treatment · A decrease in the exacerbation rate
Omalizumab	Bousquet J et al., 2021, ( 85 )	16 wk.	· GETE scale: excellent or good response to treatment (primary) · Lung function, the annualized rate of severe exacerbations, OCS use, PROs (ACQ, ACT, AQLQ), HCRU, and school or work absenteeism
Anti-IgE AntiIL5/IL5R AntiIL4R α	Abbas F et al, 2021, ( 86 )		· ≥50% reduction in clinically significant exacerbations · ≥50% reduction in mOCS dose · ≥120 mL increase in FEV1 · ≥ 3-point increase in ACT score
Mepolizumab	Kavanagh JE et al., 2020, ( 87 )	16 wk. 24 wk. 52 wk.	· ≥50% reduction in the annualized exacerbation rate · ≥50% reduction in daily prednisolone (or equivalent) dose (for patients whose condition required mOCS)
Mepolizumab	Fong WCG et al., 2021, ( 88 )	12 months	· had the top quartile of percentage reduction in mOCS dose while having a synchronous reduction in exacerbations or · if not on mOCS, had the top quartile of percentage reduction in exacerbations
Mepolizumab	Harvey ES et al., 2020, ( 89 )		· ≥0.5 reduction in the Asthma Control Questionnaire (ACQ)-5 score from baseline or · ≥25% reduction in the maintenance oral dose of corticosteroid from baseline and no deterioration in the ACQ-5 from baseline
Mepolizumab, reslizumab	Mukherjee M et al., 2020, ( 61 )	Four months	Suboptimal response: · failure to reduce maintenance corticosteroid by 50% · failure to reduce ACQ-5≤1.5 · failure to reduce exacerbations by 50% plus persistence of sputum eosinophils> 3% or blood eosinophils ≥400 cells/µL
Dupilumab	Rabe KF et al., ( 90 )	24 wk.	· The proportion of patients with a reduction from baseline of at least 50% in the oral glucocorticoid dose · The proportion of patients who had a reduced oral glucocorticoid dose to less than 5 mg daily · The annualized rate of severe exacerbation events (defined as events leading to hospitalization, an emergency department visit, or treatment for ≥3 days with systemic glucocorticoids at ≥2 times · The absolute change from baseline in the FEV1 before bronchodilator use at weeks 2, 4, 8, 12, 16, 20, and 24 · The change from baseline in the ACQ-5 score at week 24
Dupilumab	Numata T et al., 2022, ( 91 )		· ≥50% or greater decrease in exacerbations · ≥50% or greater decrease in the OCS maintenance dose, or · ≥3 improvements in the ACT score
Dupilumab	Dupin C et al., 2020, ( 92 )	12 months	· A patient with an excellent/good symptom score (1 or 2) with dupilumab treatment

GETE: Global evaluation of treatment effectiveness, AQLQ: Asthma quality of life questionnare, ACQ: Asthma control questionnare, ACT: Asthma control test, OCS: Oral corticosteroid, PRO: Patient reported outcome, HRCU: Healt care resource utilization, FEV_1_: Forced expiratory volume in the first second

It has been found that discontinuing biologics before five years in patients with a good response to omalizumab may increase the risk of relapse of uncontrolled asthma ( 97 , 98 ).

We also discontinue the use of omalizumab in patients who started treatment for severe allergic asthma and exhibit great response to the biologic after five years ( 99 ). Mepolizumab is a newer biologic than omalizumab, no trials have been conducted to compare early discontinuation with delayed discontinuation in patients who have responded well to treatment. However, it was observed that the recurrence rate after discontinuation was higher after one or two years in those who responded well to the treatment; therefore, longerterm use was recommended. ( 96 ). Since reslizumab, benralizumab, dupilumab and tezepelumab approved later than omalizumab and mepolizumab, time and further studies are required to determine the duration of treatment in patients with good responses to these biologics.

On the other hand, it is also important to carefully determine the optimal criteria for discontinuing biologics using comprehensive assessment tools for asthma control. In patients with inadequate asthma control and residual airway inflammation, discontinuing biologics may worsen asthma control if termination criteria are not stringent ( 95 ). Strict criteria for biological agent discontinuation, while limiting the number of patients who meet the criteria, would result in reduced rates of worsening asthma outcomes after treatment termination ( 8 ). In conclusion, studies on biologics discontinuation suggest that biologics discontinuation is a suitable option in cohorts of patients with SA who have reached a well-controlled status, such as superresponders. At this point, super-responder criteria and standardization are critical. Unfortunately, there is currently no consensus on these criteria ( 93 ). However, factors such as the absence of asthma symptoms, no asthma exacerbation in the previous year, no OCS requirement, suppressed T2 inflammation as measured by blood eosinophil count and FeNO level, and control of allergic comorbidities can be used to assess response. Super-responder criteria are discussed in detail in the following section.

############ Discontinuation criterias of biologics

Studies evaluating super-responders to biologics including omalizumab, mepolizumab, benralizumab, and reslizumab have been published ( 39 , 70 , 87 , 89 ). The super-responder criteria and predictive factors defined in these studies are shown in Table 5.

The proportion of super-responders among patients who receive mAbs is 24-39% ( 87 , 89 ). Another study

**Table 5:** Initial response criteria for biological agents

Biologic	Study	Super-responder criteria	Predictive factors
Mepolizumab	Kavanagh JE et al., 2020, ( 87 )	· Exacerbation-free at one year and · off mOCS	· Lower BMI · CRSwNP · Lower mOCS · Lower ACQ-6 score
Mepolizumab	Harvey ES et al., 2020, ( 89 )	· Top 25% of ACQ-5 responses from baseline or · Well-controlled asthma symptoms (ACQ-5< 1.0)	· Later age of asthma onset · High blood eosinophil levels
Benralizumab	Kavanagh JE et al., 2021, ( 39 )	· No exacerbations and · No mOCSs for asthma at 48 wk.	· Lower mOCS · Adult-onset asthma · CRSwNP · Higher blood eosinophil · Higher FEV1
Anti IL5/Anti IL5Rα	Eger K et al., 2021, ( 70 )	· Complete control of asthma after two years of anti-IL-5 treatment · No chronic OCS use, no OCS bursts in the past three months · ACQ< 1.5, FEV1≥ 80% predicted · FENO< 50 ppb · Complete control of comorbidities	· Shorter asthma duration · Higher FEV1 · Adult-onset asthma · Absence of nasal polyps · Lower body mass index

ACQ: Asthma control questionnare, OCS: Oral corticosteroid, FEV1: Forced expiratory volume in the first second, CRSwNP: Chronic rhinosinusitis with nasal polyp, BMI: Body mass index, FeNO: Fractioned exhaled nitric oxide.

reported the rate as 39% ( 39 ). However, Eger et al. reported that only 14% of patients who were treated with anti-IL5/anti-IL5Rα mAbs (mepolizumab, benralizumab, and reslizumab) met the superresponse criteria. The authors reported that the low rate of patients with super-responders to biologics might be due to stricter criteria than in other studies, implemented to lower the risk of worsening of asthma after treatment discontinuation ( 70 ). Hamada et al. also suggested using strict criteria similar to the super-responder criteria defined by Eger et al., but also emphasized the necessity of validation studies ( 8 ). Given all of this, we believe the superresponder criterion should be standardized and applicable to daily practice ( 93 ). In our clinic, we discontinue mAb therapy after five years in patients who have a very good treatment response to omalizumab and mepolizumab and continue their follow-up. Our super-responder criteria for omalizumab and mepolizumab are as follows: patients who do not have a history of exacerbations requiring the use of systemic corticosteroid in the last year, patients who have a final GINA symptom control score of 0 (or ACT score of 25) and no OCS dependency ( 93 , 99 ).

Being a super-responder to biologics is defined differently in different studies. As a result, the proportion of patients who are super-responders to biologics varies between trials. Recently, an international consensus on the definition of superresponder has been developed (100). When considering discontinuation mAbs in patients with SA receiving biologics, biologic super-responders are expected to be the strongest candidates. However, the varying definitions used for super-responders can make identifying suitable patients challenging. Furthermore, the findings of these studies indicate that not all super-responders are suited for discontinuing biologics because some have reported impaired asthma control after mAb discontinuation ( 8 ). In other words, there is a possibility that asthma control may worsen following mAb discontinuation in super-responders. For this reason, it should be remembered that patients who are super-responders and whose mAb therapy has been discontinued may have the potential to restart biologics, which should be evaluated during follow-up.

############# Efficacy after discontinuation of biologics

A few studies have been published on the discontinuation of biologics in SA. The first of these studies was the XPORT study, which evaluated the effects of discontinuing omalizumab after long-term therapy ( 94 ). This study has introduced two serious situations regarding the discontinuation of biologics. First, it was shown that approximately half of the patients whose biologics were discontinued had their asthma still well controlled, providing essential data on the prolonged efficacy of omalizumab. Second, patients without exacerbations after discontinuation had lower peripheral eosinophil counts during mAb and did not show an increase in FeNO levels compared to those with exacerbations. This suggests that suppressed T2 inflammation may be a predictive indicator for the decision to discontinue treatment. Patients who were treated with omalizumab for five years and were super-responders were included in our study to assess the effectiveness of the drug following the end of treatment. We have also suggested that one of three patients was re-treated with omalizumab due to loss of asthma control after discontinuation of the treatment ( 99 ).

An open-label prospective study also investigated the efficacy of omalizumab for four years after discontinuation of omalizumab in 49 patients with SA. In this study, the effects of long-term omalizumab therapy were shown to persist for at least four years in 60% of patients after discontinuation of treatment. Although the difference was not statistically significant, exacerbations were reported to be more frequent after treatment discontinuation in patients with chronic rhinosinusitis, NP, and NSAID intolerance. This finding suggests that comorbidities may be potential indicators of failure after treatment discontinuation ( 101 ).

The COMET trial compared stopping versus continuing long-term mepolizumab therapy in SEA ( 96 ). After the discontinuation of mepolizumab, the increase in asthma exacerbations was relatively low (61% in the discontinuation group, 47% in the continuation group), and severe exacerbations did not increase in the mepolizumab discontinuation group. No significant worsening of asthma symptoms and respiratory function was demonstrated even one year after discontinuation of treatment.

An observational study evaluated efficacy after discontinuing biologics (omalizumab, dupilumab, mepolizumab, benralizumab, and reslizumab) and found a 50% or greater increase in failure, which was defined as exacerbations requiring systemic corticosteroid administration and/or hospitalization or emergency room admission. The failure rate was 10.2% in those who discontinued treatment and 9.5% in those who continued. This result supports the view that the prolonged effect of a biological agent may continue after discontinuation in patients with SA. However, this study has several limitations, including its design as an observational database research and its lack of data on asthma symptoms and pulmonary function ( 105 ).

As a result, after stopping treatment with a biologic, asthma control may continue in some patients, while it may deteriorate in others. It appears that fewer asthma symptoms, suppression of T2 inflammation (low blood eosinophil count and/or FeNO level), and control of asthma comorbidities may be associated with the successful discontinuation of biologics. However, more research is required to identify which patients are appropriate for treatment discontinuation as well as potential predictors of continued asthma control after discontinuing treatment. In addition, the criteria for super-response to biologicals need to be standardized to identify predictors of successful discontinuation of biologics.

############## Switching Biologics in Severe Asthma

The availability of several mAb options for the eosinophilic phenotype combined with the frequent overlap of different asthma endotypes in the same patient provides clinicians with an opportunity for an alternative mAb in cases where the initial choices do not result in optimal therapeutic efficacy ( 103 , 104 ).

############### Switch between anti-IL5/IL5Rα biologics

Clinical responses to anti-IL5/IL5Rα mAbs may not be the same in all patients. While some patients have complete asthma control (super-responder) with the addition of these biologics, some continue to experience partial-responder symptoms or no improvement. In rare cases, clinical worsening may occur (non-responder) ( 39 , 61 , 89 ). Approximately 24% to 42% of patients with SEA have partial or no response to anti-IL5/IL5Rα treatments (39,89). The mechanisms underlying these different responses are not yet exactly known. Table 6 summarizes the viewpoints presented regarding the likely causes of the variability observed in responses to anti-IL5/ IL5Rα therapies ( 60 , 70 , 105 , 116 ).

There is currently limited data on the efficacy of switching anti-IL5/IL5Rα agents. When compared to reslizumab and benralizumab, the effect of mepolizumab SC 100 mg on airway eosinophilia appears to be rather limited when examined using induced sputum ( 70 , 117 , 118 ). Airway mucosal eosinophils are reduced by approximately 96% in bronchial biopsies of asthmatic patients after three consecutive subcutaneous administrations of benralizumab ( 118 ). Similarly, a weight-adjusted dose of reslizumab can significantly reduce sputum eosinophilia by approximately 91% ( 70 ). Therefore, the responses to different anti-IL5/IL5Rα mAbs in the same patient may differ in SEA ( 70 ). In a study of more than 250 patients with SA treated with mepolizumab or reslizumab, most suboptimal responders had elevated IL5 in their sputum. This suboptimal response was thought to result from inadequate neutralization of IL5 in the airway ( 61 , 115 , 119 ). Because of the different responses to anti-IL5/IL5Rα treatments and the possible mechanisms mentioned above, in real-life, clinicians may switch to another anti-IL5/IL5Rα mAb in patients with partial or no response to an anti-IL5/IL5Rα mAb to achieve optimal disease control ( 70 ).

In a real-world study evaluating the long-term (minimum two years) use of anti-IL5/IL5R mAbs in SEA and switching between these biologics, 59 percent of patients reported no difference between anti-IL5 biologics. It was reported that 34% of these

**Table 6:** Possible reasons for the observed heterogeneity in responses to anti-IL5/IL5R treatments ( 60 , 70 , 105 , 116 )

Individual differences in the pharmacokinetics of the biological agent
Antidrug antibodies against biological agent
Degree of remodelling of the upper and lower airways
Activation of non-IL5-mediated inflammatory pathways
Other cytokines related to ILC2 biology
Differences in the blocking effect of biologics on IL5 signaling
Comorbidities that may lead to asthma-like symptoms, e.g., dysfunctional breathing, obesity, deconditioning, or cardiovascular disease

patients were switched to another anti-IL5/IL5R, and 7% had switched to two different biologics during follow-up (70). Persistent asthma or sinonasal symptoms, including exacerbations, were identified as the most common cause of switching biologics (%58). This was followed by failure to reduce or stop OCS (28%) and permanent airflow limitation (17%). Only a small percentage were switched due to adverse drug reactions (5%).

A small case series of three patients with eosinophilic asthma and suboptimal response to mepolizumab also demonstrated significant clinical improvement after switching to benralizumab ( 120 ). A short study of patients with eosinophilic asthma who switched from mepolizumab to benralizumab without a washout time found that all study results improved significantly (121). In another study involving a small number of patients, lung function in OCS-dependent patients with blood eosinophilia >300 cells/µL and sputum eosinophilia >3% and poor response to mepolizumab 100 mg SC every four weeks when switching to weight-adjusted IV reslizumab and improved asthma control were reported ( 60 ).

Although these observations support the hypothesis that non-response to mepolizumab in patients with eosinophilic asthma does not prevent subsequent response to reslizumab and benralizumab, studies involving more extensive series of patients are needed as these are case reports and studies involving a limited number of patients.

################ Switching from anti-IgE to anti-IL5/IL5Rα therapies

Switching from omalizumab to mepolizumab, reslizumab, and benralizumab has been investigated, primarily due to the differences in time points at which biologics were approved for therapy ( 122 , 123 , 124 , 125 ). A study evaluating the switch from omalizumab to reslizumab reported a significant increase in median ACT scores and a decrease in OCS requirement when switching to reslizumab. The authors suggested that reslizumab may be an effective and safe option for patients with SEA who have previously failed with omalizumab ( 126 ).

In the OSMO study evaluating the patients who switched from omalizumab to mepolizumab, patients with SEA who were treated with omalizumab (for at least four months) and whose asthma was not under control were switched to mepolizumab for 32 weeks ( 69 ). Following the switch, significant improvements in asthma control, quality of life questionnaires, lung function, and exacerbation rates were reported in patients with uncontrolled SEA. However, this study had some limitations. These limitations include the single-arm and open-label study design, monitoring endpoints for only up to 32 weeks rather than 12 months, and the first indications for prescribing omalizumab for all patients being unknown.

To the best of our knowledge, there is no study evaluating the switch to benralizumab in patients with inadequate response to omalizumab. Only one case report reported clinical and functional improvement after switching to benralizumab in an atopic eosinophilic asthmatic patient who had an insufficient response to omalizumab ( 124 ).

################# Switching from an anti-IgE or anti-IL5/IL5Rα to anti-IL4Rα biologic

In a recent study, an improvement in asthma control, a decrease in exacerbations, and a decrease in the need for systemic corticosteroids were shown by switching to dupilumab in patients who did not respond adequately to anti-IgE or anti-IL5/IL5Rα treatments. In this study, it was stated that FeNO≥ 25 ppb could be used as a potential biomarker to predict response when switching to dupilumab in patients who did not respond adequately to the initial biologic ( 127 ).

################## Frequency of switching biologics in severe asthma in real-life

**Figure 1:**
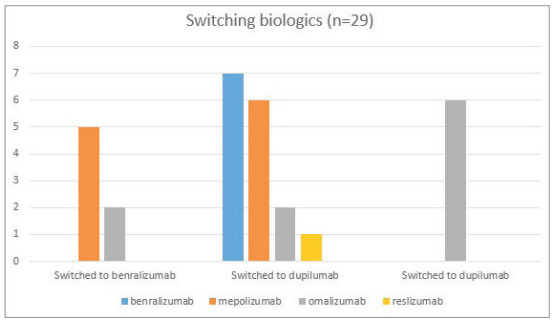
Analysis of MUC5B and TERT genes among study groups.

The outcomes of patients who did not respond effectively to the initial biologic treatment and were switched to a different mAb were examined in a realworld study ( 86 ). Figure 1 shows the agents that patients switched to/from. Approximately one in four patients were switched to another mAb due to suboptimal response to their first biologic. This finding is consistent with previous reports. Significant improvements were found in the frequency of exacerbations, maintenance of OCS dose and asthma control with the biologics patients switched to. This study also suggested that switching to benralizumab may also be effective in patients with inadequate response to mepolizumab. As a result, it has been shown that patients who do not have an optimal response to biologics can benefit from switching to a different one.

In a study involving adults with SA who were treated with biologics and enrolled in the International Severe Asthma Registry Data System (ISAR) and the CHRONICLE study in which eleven countries participated, it was reported that most of the patients included in the analysis continued their first biologics for at least six months (79%) in the follow-up, while a small portion of them was discontinued (10%) or switched (11%).

When the pre-biological characteristics of patients who continued their initial prescribed biologics were compared to those of patients who switched biologics, the switch patients exhibited higher blood eosinophil levels, OCS dependency, FeNO levels, and chronic eosinophilic rhinosinusitis ( 10 ).

As a result, when we evaluate switching between all of these biologics in general, the switch from omalizumab to other biologics appears to be more common, owing to the fact that omalizumab was the first biologic used in SA, was approved 12-15 years ago, and entered clinical use before other biologics. Omalizumab was the only treatment, especially for overlapping SA phenotypes such as atopic eosinophilic asthma. With the introduction of novel biologics, patients with this phenotype who did not respond to omalizumab or had a suboptimal response were switched from anti-IgE to anti-IL5/IL5Rα or antiIL4Rα treatment. There are currently no studies evaluating patients with this phenotype who switched to omalizumab after failing anti-IL5/IL5Rα or antiIL4Rα. It has also been observed that clinical response can be obtained when switching to another anti-IL5/ IL5Rα in patients who did not respond to the first anti-IL5/IL5Rα biologic. Aside from a poor response to treatment, the patient may have to switch from one mAb to another due to adverse drug reactions, the necessity for a more convenient dosing schedule, and patient preferences. It should also be noted that special conditions such as pregnancy, lactation, opportunistic infections, and comorbidities may require switching biologics ( 128 ).

## CONCLUSION

**Figure 2:**
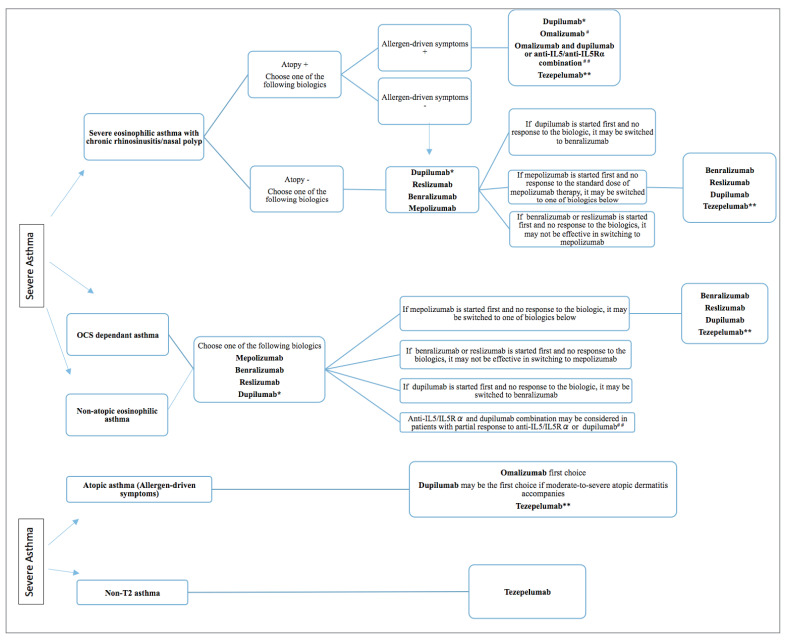
Decision tree for the biologic treatment of the severe asthma. *First option for patients who have also atopic dermatitis and high FeNO levels (≥25 ppb ), If blood eosinophils ≥1500 cells/µl, it is not recommended **Real-life studies are needed on its equality or priority over other biologics in T2 asthma. It can be considered in asthma with T2 asthma that does not respond to other biologics # If patient’s atopy status is really appropriate, given the clinical history (childhood allergic asthma, comorbidities such as allergic rhinitis, and respiratory symptoms with exposure to aeroallergens) # # Cost-effectiveness? Safety Abbreviations used: Ig: immunoglobulin; SC: subcutaneous; IL: interleukin; IV: intravenous; FeNO: fractional exhaled nitric oxide; ppb: mg:miligram; ppb: parts per billion.

The current GINA report recommends the addition of biologics as add-on therapy in step 5. However, because SA phenotypes can overlap, some patients may be candidates for multiple mAb therapies. Therefore, clinicians should make the best use of all predictive factors to identify patients who will most benefit from each available and approved treatment. Although there is increasing evidence that another biological agent should be selected for better outcomes in patients with poor asthma control, there is still an unmet need to identify and validate biomarkers that can highly predict response to different mAbs. Indeed, patients who do not reach a specific response threshold after a reasonable period of time for response evaluation (often four months) and who are eligible for one or more alternative biological agents should be given the option of switching to another biologic. However, when choosing the initial biologic, a detailed evaluation of clinical and laboratory markers that may predict the potential to benefit from the biologic may lead to fewer switches. In Figure 2, we also present the

decision tree for initial biologic selection based on severe asthma phenotypes and alternative biologics as in-house decision-making treatment based on research conducted to date.

The discrepancy between known T2 biomarkers and the clinical response to mAb in some patients suggests that the underlying inflammatory pathways may be much more complex than expected. Targeting one mechanism may not be sufficient, and there may be multiple therapeutic potentials in selected patients.

There is no consensus on when to discontinue mAbs in SA patients with good response to treatment. We recommend using mAbs for at least five years as early treatment termination may potentially deteriorate asthma control. Standardizing the super-responder criteria for treatment would allow for more consistent studies on the subject as well as a more precise determination of the time to discontinue treatment. More research and consensus reports are required in this context.
